# Network characteristics and patent value—Evidence from the Light-Emitting Diode industry

**DOI:** 10.1371/journal.pone.0181988

**Published:** 2017-08-17

**Authors:** Way-Ren Huang, Chia-Jen Hsieh, Ke-Chiun Chang, Yen-Jo Kiang, Chien-Chung Yuan, Woei-Chyn Chu

**Affiliations:** 1 Department of Biomedical Engineering, School of Biomedical Science and Engineering, National Yang-Ming University, Taipei, Taiwan; 2 Department of Business Administration, National Yunlin University of Science and Technology, Yunlin, Taiwan; 3 School of Economics and Management, Wuhan University, Wuhan, China; 4 Department of Business Administration, CTBC Financial Management College, Tainan, Taiwan; East China University of Science and Technology, CHINA

## Abstract

This study proposes a different angle to social network analysis that evaluates patent value and explores its influencing factors using the network centrality and network position. This study utilizes a logistic regression model to explore the relationships in the LED industry between patent value and network centrality as measured from out-degree centrality, in-degree centrality, in-closeness centrality, and network position, which is measured from effect size. The empirical result shows that out-degree centrality and in-degree centrality have significant positive effects on patent value and that effect size has a significant negative effect on patent value.

## Introduction

Light-Emitting Diode (LED) is a kind of material that transforms electric energy to luminous energy. Compared to the traditional illuminant such as electric incandescent lamp and neon lamp, LED products which has the advantages of energy conservation, small volume, strong shock resistance ability, high-brightness, eco-friendly, long operating life are attractive to the world’s attention particularly in display and lighting fields. In order to maintain competitiveness in LED industry, it is important for leading manufacturers to master core technologies of LED products. In this case, owing the exclusive right of patent is an appropriate mean to build up legal entry barriers and reduce competitions. Therefore, acquiring patents is the top priority for LED manufacturers to prevent other rivals entering relevant field of technology.

Although prior studies provided many effective methods for evaluating patent value [1–12], the chosen indicators create limitations to using patent characteristics to predict the possibility of litigation or to evaluate patent value. This result occurs because, firstly, all indicators merely statically evaluate and explain patent value and litigation. Only when patent characteristics are identified can the true value of a patent be evaluated. Secondly, although forward citations are viewed as an effective indicator to predict potential litigation [11, 13], we find that patents with fewer or no forward citations also become involved in litigation, indicating that the patent characteristic perspective is insufficient and we require a more effective method.

This study proposes a different angle for evaluating patent value, namely by regarding patent value as a combination of static characteristics and the mainstream indicators of social network analysis. On the one hand, the majority of patent static characteristics are fixed in their application, which means that these characteristics are controlled by the patent’s owner. On the other hand, a citation relationship is formed through forward and backward citations, indicating that the patent is not controlled by the owner. A patent that takes an important position in other citation relationships implies that the patent has a higher probability of gaining and controlling technology and knowledge and of having an important influence on subsequent patents. In other words, the patent is valuable.

This study applies social network analysis to carry out the research purpose. To develop a patent citation network, this study regards the patent as a node and citations as the connections between the nodes. This study provides a mode of thinking for companies using social network analysis, offers a more rational way to evaluate patent value, and constructs an effective early warning mechanism for patent litigation.

## Literature review and hypothesis development

### The determinants of patent value

Prior researchers developed ways to evaluate patent value, primarily using patent characteristics (static attributes) as the relevant discussed determinants. Prior studies primarily categorized the independent variable into four different classes: patent characteristics (number of backward citations, international patent classification (IPC) categories, and number of inventors, among others), patent ownership (co-application and cross-border ownership, among others), insider information (motivation for application and background of the invention, among others), and filing strategies (number of patent claims and patent priority, among others) [4]. From these variables, we obtain various valuable information such as technological importance [14], the existing technological background, the linkage between innovation and basic research [15], technological scope [16], research effort [17], the legal breadth of the protection [18], and so on. Therefore, researchers conducted various empirical studies to predict a patent’s potential value. Allison, Lemley [8] found that valuable patents contain more claims, forward citations, and backward citations. Similarly, Fischer and Leidinger [2] found that forward citations and family size are significant indicators for patent value. Allison, Lemley [19] discovered the features of litigated patents and concluded that litigated patents are of higher value than patents that have not been litigated. Bessen [6] found that a litigated patent is worth nearly six times as much as non-litigated patent. Chang, Yang [20] derived a new variable called the earn plan ratio, indicating the extent to which a company’s internal strategies affect its external reputation. They also built a regression model by using compensation for damages as a dependent variable and demonstrated that patent family strength reflects the value of the patent itself. Reitzig [9] analyzed the patents in semiconductor industry and found that knowledge of technical importance, the position of the patent in a portfolio, learning value for competitors through disclosure, and the difficulty in inventing have significant positive effect on patent value. Wu, Chang [1] applied logistic regression model to analyze sample of LED industry, the results demonstrated that patent value has positive relationship with patent number of patent claims, number of backward citations, patent family depth, and earn plan ratio. The foregoing studies consistently demonstrated that forward citations, patent family, renewals, legal disputes, and application strategies are positively related to patent value. However, the relationship between the other determinants and value is blurry [4]. This phenomenon demands further studies that focus on a new perspective.

### Network characteristics indicators

The network centrality indicators and position indicators have been the mainstream indicators in social network analysis. Centrality analysis in sociological literature dates back to decades [21]. Freeman [22] can be considered a pioneer when he proposed dimensions of centrality in a social network, which have been developed into degree centrality, closeness centrality and betweenness centrality. Lately, many researchers began to focus on social network analysis indicators in different fields. For example, Burgers, Hill [23] analyze relationships between patent centrality indicators and strategic alliances. Badar, Hite [24] found that authors having more coauthors will have high degree centrality bringing benefits of knowledge sharing to these central authors who can use this knowledge to enhance their research performance in the context of co-authorship networks. Perry-Smith and Shalley [25] elaborated high closeness centrality for an author in the co-authorship network specifies that he has access to large portion of the knowledge flowing in the network.

Given the interdisciplinary use of social network analysis, researchers also combined the method with patent analysis to offset the disadvantage of traditional patent analysis methods. Chang, Lai [26] proposed a method for exploring the fundamental patent indicator and evaluating the relationship between fundamental patents. They also classified fundamental patents and elaborated on technology diffusion by developing a patent citation network to analyze patent business methods. Social network analysis not only assists in identifying the relationships between patents but also in identifying the relationships between patent owners. Kim and Song [27] connected the two sides involved in a lawsuit by building a patent litigations network and dividing the companies into four classes on the basis of the level of initiative and passivity according to the centrality of companies. They then judged the roles played by the companies in the specific technology field accordingly, as well as their future development direction.

Burt [28] demonstrates that brokerage and closure positions act as complements in controlling a network. Being in a brokerage position enhances an actor’s ability to broker beyond its group, but the closure position affects the closeness within a group. According to social network analysis, von Wartburg, Teichert [29] found that a patent’s position within a patent citation network can be a critical factor that facilitates the dissemination of technological knowledge. Blind, Cremers [30] thought that patents in a brokerage position can be viewed as a strategic tool for quickly blocking competitors entering the market, as these patents are in an important position to connect with related patents. Wang, Chiang [31] studied the effects of brokerage or closure positions on patent quality in evolving technology by drawing a patent citation picture that regards the patent as a node and a citation as a connection.

### Network centrality and patent value

Network centrality is an indicator that evaluates the ability of a node to access and control resources, which means that a node with higher network centrality is more directly or indirectly connected to, and has greater influence over, other nodes. Therefore, a node with higher centrality occupies higher relative importance, affects some nodes more, and is less affected by other nodes. To evaluate centrality, we introduce two widely used indicators in social network analysis, explain the indicators, and develop the hypotheses.

Degree centrality evaluates the number of other nodes that are directly connected to one node, reflecting the local centrality of the node. Therefore, nodes with higher degree centrality are connected to more nodes and possess more informal rights and influence. Consequently, such nodes exert influence on the entire network through the nodes connected to them and have a greater chance of gaining access to resources.

Patent applicants need to list the cited patents or scientific literature, and such backward citations constitute the technical basis of the patent. Connecting all of the patents in order of citation relationship enables us to draw a dynamic picture of the evolving technology network. Because a patent citation network is an ordered network, it requires to simultaneous consideration of in-degree centrality and out-degree centrality.

Out-degree centrality represents the number of relationships that started from one node to the other nodes and indicates the number of patents cited by one patent or one patent’s backward citations. Generally, because fundamental patents focus on basic research, they do not have many patents or scientific studies to cite, resulting in lower out-degree centrality. In contrast, a backward citation evaluates the extent of technological spillovers [32–34]. A patent based on a large number of previous studies can fuse the previous technology knowledge and cover multiple technological fields or dig deep into a specific technology field. Such a patent possesses higher technology breadth or depth. Therefore, a patent with higher out-degree centrality has a higher technological degree of fusion. Allison, Lemley [8] showed that litigated patents have many more citations than patents that are not litigated. In terms of averages, litigated patents cite an average of 14.2 patents, and non-litigated patents cite an average of 8.6 patents. Thus, patents with high out-degree centrality could have higher value. Therefore, this study proposes the following hypothesis.

**H1**: **Litigated patents have higher out-degree centrality than patents that have not been litigated**.

In-degree centrality represents the number of relationships that one node obtains from the other nodes; this figure indicates the number of patents that have cited this patent or the number of forward citations. Only when the technology knowledge contained by a patent is necessary or provides reference for subsequent innovations is this patent cited by a subsequent patent. Therefore, studies on forward citation usually view forward citations as the evaluation of patent technological quality and the economic value of one technique to another [10, 12, 32, 35, 36]. A patent that has a large number of forward citations reflects the concept that its unique technique is core or critical in this technology field, subsequently attracting a large number contenders. Therefore, patents of higher in-degree centrality have higher value in terms of technique and economics. Therefore, we propose the following hypothesis.

**H2**: **Litigated patents have higher in-degree centrality than those that are not litigated**.

Closeness centrality computes the sum of all of the shortest distances between one node to all other nodes, and then reflects the extent to which an actor can avoid being controlled through an explanation of efficiency and independence [22]. Efficiency shows the fewest number of steps that an actor will take to get to another actor, whereas independence indicates that the actor can connect with others with weaker dependence on intermediary personnel. Hence, higher closeness centrality means that an actor has higher efficiency and independence. Because a patent citation network is an ordered network, in-closeness centrality and out-closeness centrality must be distinguished. The meaning of in-closeness centrality is the reciprocal of the sum of the shortest distance for a patent to be cited indirectly or directly and reflects the extent to which a patent affects subsequent patents without interference from the mediator. Out-closeness centrality evaluates the reciprocal of the sum of the shortest distance for one patent to cite all patents that it needs, thus reflecting its ability to absorb technical knowledge from previous patents. Because patent value mainly consists of technological value and economic value, technological value is a reference basis for subsequent patents and economic value represents the profitability of patent commercialization. However, patent profitability largely depends on the technological value reflected in forward citations. Therefore, this study only considers in-closeness centrality. As previously noted, patents with higher in-closeness centrality have a more significant effect on subsequent patents. Therefore, this study proposes the following hypothesis.

**H3**: **Litigated patents have higher in-closeness centrality than those that are not litigated**.

### Network position and patent value

Social capital can be acquired from two types of network positions: structural holes and network closures. Participating and controlling information diffusion are the basis for social capital brought on by structural holes. The theory of the structural hole contends that social capital is a kind of intervening opportunity, a non-redundant relationship between two relevant privies. A structural hole provides a cushion in a network, whereas individuals around structural holes cycle through different information flows, enabling structural holes to bridge the different information flows and control people bilaterally. For example, a network has three nodes, A, B, and C, with B and C connected to A, whereas B and C are not connected to each other. In this configuration, A occupies the central position, namely the structural hole position, and B and C can only be associated through A. In other words, the node in the structural hole position possesses better mediation, which puts it on the critical path that connects different nodes to more effectively control the flow of resources and information [22].

As indicated by Burt [28], nodes located on the structural hole disseminate information or allocate resources among the different groups or nodes, creating communication and exchanges between nodes that are not directly connected. Such interaction makes it possible for the node on the structural hole to create value. In patent citation networks, mediators transfer technological knowledge to patents in different fields. By connecting patents in different fields, the mediators can easily create new techniques and promote technological development. Additionally, because mediators play an important role in connecting relevant patents, they can be deemed as strategic tools to quickly block contenders from entering the market [30]. Therefore, patents in the structural hole tend to have greater value.

Network closure is often highly correlated with network density. The definition of network density is the average level of interaction among members of the network, and higher density means a higher number of connections among members [37]. Network closeness reaches the highest level when every node is connected to one another, either directly or indirectly. With nodes closely connected and actors frequently exchanging information in a closed network, a closed network reduces the heterogeneity in the group, which promotes reliance and collaboration, strengthens knowledge sharing, and improves group performance. All actors establish their own reputations in the network, which more closely links them together and forms a closed network [31]. Therefore, frequent contact among network members makes it easier to produce similar views, establish a common belief, and reach an agreement, leading to stronger cohesion and a more profound effect from group norms on the network. In turn, this phenomenon reduces the heterogeneity of network members. In a patent citation network, technological knowledge of highly concentrated and overlapping information communication helps to deeply comprehend and absorb specific technological knowledge, thus deepening R&D and continuously improving existing technologies. Moreover, a closed network structure can efficiently reduce technological risk, increase cooperation opportunities with analogous technology, and improve the communication efficiency of homogeneous technologies.

However, closed networks can limit one node from relationships with adjacent nodes and expose one node to the risk of being controlled and replaced. More specifically, assume that nodes A, B, and C are connected to one another, meaning that the relationship between A and B contains redundant information. This relationship indicates that B may be connected to C by A, whereas B and C can directly exchange information, thus weakening the control by A over B and C. That is, networks of high closeness restrain A to the relationship of A, B, and C, giving the network higher embeddedness. Therefore, high patent closeness indicates that the patent focuses on a certain specific field, causing redundant information between the patent and patents adjacent to it. Given the homophyly with adjacent patents, subsequent patents need not cite this patent, and the adjacent patents can potentially replace it. Thus, the patent may no longer have a significant impact on subsequent patents.

Structural hole and network closeness are two sides of the same network position, and we can use effect size to evaluate the position of the node. The definition of effect size is the difference between individual network scale and network redundancy, i.e., the actual size of a non-redundant network, reflecting the non-redundant information that exists in the relationship between the node and adjacent nodes [38]. A larger effect size of the node results in a lower degree of duplication and a stronger intermediary. Therefore, this study proposes the following hypothesis.

**H4**: **Litigated patents have a higher network position than those that are not litigated**.

## Methodology and measurement

### Sample and data collection

The data for this study come from the Thomson Innovation database. We retrieve all of the literature information on U.S. authorized patents up to May 31, 2011. Technology fields retrieved include epitaxial growth, LED chip making, and LED chip packaging for end product application technology. After a preliminary retrieval, 40,330 patents were selected. Through manual screening, we obtained 7,164 patents belonging to this study’s relevant technology fields. To confirm appropriate LED technology fields, this study engaged in in-depth interviews of ten senior experts who participated in R&D projects and who have more than ten years of experience in the LED industry. (S1 File) Through the interviews, keywords for retrieval and analysis data were determined. Further, we removed isolated patents that are not cited or are cited by other patents, which left 4,823 suitable patents. We the adopted Westlaw’s database to check by patent number whether or not the sample patents were litigated. Finally, we obtained a sample set of 59 litigated patents and 4,764 patents that were not involved in litigation. (S1 Data)

### Ethics statement

No personal or identifying information was collected from interviewees, and so written consent was not required. The ethics committee approved the verbal informed consent procedure.

### Measurement

Litigated versus Non-litigated patents: A dependent variable is used; this is a categorical variable and is coded 1 if the patent has ever been involved in litigation and 0 if the patent has never been involved in litigation.

Prior to litigation, a patentee and a non-patentee hold different expectations of the potential outcome. A successful case for the patentee, with the patent being upheld, is likely to mean continued profits and possibly the payment of damages. If the patentee loses in court, there is a loss of the exclusive rights to the patented technology and this is likely to result in lower profitability and further legal fees. To avoid infringement litigation that might result in the above outcomes, patentees may authorize competitors to use a patented technology through a license. Generally, both patentees and non-patentees independently evaluate whether a lawsuit is worthwhile and likely to be successful and the expectations of winning the patent case will determine whether or not litigation takes place. In such circumstances, when the expectations of winning held by both sides are close, they are more likely to reach a settlement without resorting to the courts [5].

Profit margins are highly correlated with the value of a patent and a patent with a high value often results in greater profits to a company. Thus the value of a patent has a positive impact on whether patent litigation is pursued [11, 39]. As the value of a patent increases, this increases the probability of infringement, as well as the likelihood of litigation as opposed to settlement. In turn, litigation can affect the value of a patent. If litigation is likely to fail, the prospect of losing can significantly reduce the value of a patent. Conversely, the value of a patent is increased if a patent case is won and this also helps to protect and maintain a company’s market share. In recent years there had been a rapid rise in globalization and companies have taken advantage of the legal value of patents. This has resulted in the development of legitimate commercial strategies aimed at preventing potential contenders from entering the market. From a legal perspective, successful patent litigation can be used as a measure of the value of patents [40] and based on this premise this study uses “litigated patent” as the proxy variable for “patent value.” The data used for this study was obtained from Westlaw, a online legal research service, and means that there is a high degree of certainty as to whether or not a patent has been litigated on.

Out-degree centrality (OutDegree): the number of times that patent *i* cites other patents or the number of patent *i*’s backward citations; defined by *d*(*i*) = Σ_*j*_ *m*_*ij*_, if patent *i* cites patent *j*, *m*_*ij*_ = 1.

In-degree centrality (InDegree): the number of times that patent *i* is cited by other patents or the number of patent *i*’s forward citations; defined by *d*(*i*) = Σ_*j*_ *m*_*ji*_, if patent *i* is cited by patent *j*, *m*_*ji*_ = 1.

In-closeness centrality (InCloseness): reciprocal of the sum of the shortest distance taken by all other patents to cite patent *i* directly or indirectly; defined by *c*(*i*) = Σ_*j*_ *d*_*ji*_, where *d*_*ji*_ is the shortest distance from patent *j* to *i*.

Effect size (EffSize): the difference between individual network scale and network redundancy, or factors other than network redundancy; defined by *ES* = Σ_*j*_(1 – Σ_*q*_*p*_*iq*_ *m*_*jq*_), *q* ≠ *i*, *j*, where *j* represents all nodes connected to *i* and *q* represents the nodes except for *i* and *j*; *p*_*iq*_ *m*_*jq*_ represents the redundancy between patent *i* and *j*, and *p*_*iq*_ represents the proportion of the relationship input from patent *i* to *q*.

Patent inventors (PatInventor): number of inventors; reflects a patent’s technological complexity and research effort needed. Generally, a larger number indicates higher technological complexity and greater research effort, which makes the patent more likely of higher value. This study defines patent inventors as the number of patent inventors. The data are from the Patent Full-Text and Image Database established by the USPTO.

Patent technology scope (PatTechScope): measures the reach of the field of patent technology, and is primarily reflected in the number of technology classifications, such as IPC (International Patent Classification) or UPC (United States Patent Classification). Patents with a higher number of technology classifications have broader technology scope. Prior studies indicated that patent technology scope is positively correlated with patent value: more scope indicates being more involved and a higher number of valuable inventions being created [3, 16, 41]. In this study, technology scope is defined as the sum of a patent’s second-order sub-classification number without repetition, or the sum of the four-digit IPC code.

Patent claims (Claim): this variable defines the scope of the protection covered by the patent and one or more patent rights under certain conditions, including independent and dependent claims. Using the patent application, patent claims state the technical features and whether or not the patent has been infringed. For the patentee, a higher number of claims means broader patent protection [7], which assists companies in identifying potential infringers or authorizers in certain specific fields. Lanjouw and Schankerman [11] found that if patent claims increased by 10%, the number of patent litigations in their sample increased by 1.4%. Therefore, more patent claims represent broader protection scope, which increases the value of a patent. The number of patent claims is defined as the sum of the independent and dependent claims. The data are from the Patent Full-Text and Image Database by the USPTO.

Patent family (PatFamily): a collection of applications of the same patent in different countries or a collection of different subsequent patents from the same patent; indicates the company’s attention to technology and the market. By applying patents in several important countries for protection, the company can erect barriers for opponents that attempt to imitate its technology and undermine its competence and market share. For a company to expand the patent family through additional patent applications is more expensive. We observe that a company will invest to expand the patent family only when patent technology brings profits that are greater than the application expense. Thus, the company’s potential sales market can be predicted from the patent family layout because the quantification of the patent family reflects the importance of the technology and future markets, and patent family positively affects patent value [10, 42, 43]. The patent family value is defined as the patent amount of the focus patent. The database sampled in this study is from INPADOC (International Patent Documentation Center) at the website esp@cenet.

## Results

### Descriptive statistics and correlation matrix

The descriptive statistics and correlations matrix of this study are shown in Table 1, and include minimum, maximum, mean, and standard deviation values. The correlations matrix of this study is shown in Table 2. Table 2 shows that the probability of patent litigation is significantly positively correlated with OutDegree, InDegree, EffSize, PatTechScope, Claim, and PatFamily.

**Table 1:** Descriptive statistics.

Variables	Min.	Max.	Mean	S. D.
1. OutDegree	0	134	2.97	7.84
2. InDegree	0	292	2.97	7.36
3. InCloseness	0.021	0.028	0.021	0.0003
4. EffSize	0	288.51	5.48	10.31
5. PatInventor	1	15	2.92	1.85
6. PatTechScope	1	13	1.66	0.95
7. Claim	1	169	17.35	14.70
8. PatFamily	1	330	8.83	21.12

**Table 2:** Correlations matrix.

Variables	1.	2.	3.	4.	5.	6.	7.	8.
1. LitigatedPat	1							
2. OutDegree	0.13**	1						
3. InDegree	0.05**	0.04**	1					
4. InCloseness	0.02	-0.06**	0.64**	1				
5. EffSize	0.12**	0.74**	0.70**	0.38**	1			
6. PatInventor	0.02	0.03*	0.08**	0.05**	0.07**	1		
7. PatTechScope	0.03**	-0.02	-0.05**	-0.07**	-0.05**	0.04**	1	
8. Claim	0.05**	0.06**	0.16**	0.14**	0.14**	0.11**	0.07**	1
9. PatFamily	0.09**	0.04**	0.22**	0.19**	0.17**	0.15**	0.18**	0.20**

**p<0.05,

*p<0.1

### T-test results

This study applied a t-test to compare the mean of the litigated patents with that of the non-litigated patents, as reported in Table 3. The averages of the litigated patents are superior to those of the non-litigated patents. Regarding the mean of OutDegree, InDegree, EffSize, PatTechScope, Claim, and PatFamily, litigated patents have values that are larger than those of non-litigated patents. However, Table 3 shows that the means of InCloseness and PatInventor of litigated patents were not significantly larger than the means for non-litigated patents. Testing the mean reveals a difference between litigated and non-litigated patents.

**Table 3:** Characteristics of litigated patents and non-litigated patents.

Variables	Litigated patent	Non-Litigated patent	t-value	p-value
1. OutDegree	11.76	2.86	8.733	0.000**
2. InDegree	6.02	2.94	3.198	0.001**
3. InCloseness	0.021	0.021	1.320	0.187
4. EffSize	16.42	5.35	8.254	0.000**
5. PatInventor	3.25	2.29	1.401	0.161
6. PatTechScope	1.95	1.66	2.309	0.021*
7. Claim	23.49	17.28	3.229	0.001**
8. PatFamily	26.71	8.61	6.574	0.000**

**p<0.01,

*p<0.05

### Result of logit regression

This study uses Litigated Patent as a dependent variable, OutDegree, InDegree, InCloseness, and EffSize as independent variables, and PatInventor, PatTechScope, Claim, and PatFamily as control variables to develop a regression model. The empirical results are noted in Table 4.

**Table 4:** Result of traditional logit model and rare events logit model.

Variables	Traditional logit model	Marginal effects	Rare events logit model
Intercept	-0.73 (12.48)		-1.42 (12.45)
Independent variables			
OutDegree	0.23** (0.08)	0.003** (0.001)	0.22** (0.08)
InDegree	0.22** (0.09)	0.003* (0.001)	0.22* (0.09)
InCloseness	-219.34 (594.80)	-2.54 (6.91)	-187.42 (593.69)
EffSize	-0.21** (0.08)	-0.002* (0.001)	-0.20* (0.08)
Control variables			
PatInventor	0.03 (0.08)	0.0003 (0.0009)	0.03 (0.08)
PatTechScope	0.13 (0.08)	0.002 (0.001)	0.14 (0.08)
Claim	0.007 (0.005)	0.0000 (0.0000)	0.008 (0.005)
PatFamily	0.009** (0.002)	0.0001** (0.0001)	0.009** (0.002)
-2 Log Likelihood	584.43		N.A.
Prob > χ2	0.000		N.A.

**p<0.01,

*p<0.05.

Robust Standard errors are reported in parentheses.

The results in Table 4 indicate that, at a 5% significant level, the coefficients of OutDegree, InDegree, and EffSize are 0.212, 0.205, and –0.198, respectively. This result indicates that OutDegree and InDegree have significant positive effects on patent value, whereas EffSize has significant negative effects on patent value. Therefore, H1, H2, and H4 are supported in this study. However, in-closeness centrality has no significant effects on patent litigation probability, indicating that H3 is not supported in this study.

Because the patent has involved in litigation are extremely rare events, with a large number of nonevents (i.e., non-litigated patents) and very few events (i.e., litigated patents), it is well known that traditional logit regression models can sharply underestimate the probability of occurrence of events [44, 45]. To correct for the bias, we use the rare event logit regression (relogit) developed by King and Zeng [44] to re-estimate (using the procedure in Tomz, King [46]) the regressions for third column of Table 4 and find that there are very similar estimates of the parameters and the standard errors we report with a traditional logit regression in first column of Table 4.

The second column of Table 4 presents the estimated marginal effects of network characteristics variables and the control variables on the probability of patent litigation at the mean values of the explanatory variables. The marginal effects of these variables indicate that the change in the probability is induced if their value increases by one standard deviation. The means of the outcome variables are shown at the bottom of the table.

The results show that network characteristics matter for the probability of patent litigation, as several (OutDegree, InDegree, EffSize, and PatFamily) of them have marginal effects that are significantly different from zero. This study focus on the effects of the network characteristics. OutDegree and InDegree have a significantly positive partial effect on the probability of patent litigation. Increasing OutDegree and InDegree by one standard deviation raises the patent litigation probability by 0.3 percentage. The positive effect on the self-employment probability is explained by the positive and significant effect on the entry probability. EffSize the second largest influence on the patent litigation probability among the network characteristics. As expected, the effect is negative. A decrease by one standard deviation significantly raises the probability of patent litigation by 0.02 percentage. However, King and Zeng [44] had devised their weighting scheme of rare-events corrections. However, the results was not consistent with marginal effects that we prefer.

## Conclusions and discussion

This study applies social network analysis to evaluate patent value. It is important for practitioners in the LED industry to understand this relationship. Hence, this paper attempts to fill this research gap and reports several interesting findings.

First, out-degree centrality, which represents the number of patent backward citations, positively and significantly affects patent value. This result indicates the fusion of a patent’s technological foundation and knowledge. This study suggests that, when filing patents, companies should first fully excavate previous studies in relevant technology fields to gain a strong understanding of the development path and the cutting-edge nature of the technical field. Companies should also acquire adequate technical knowledge and build a solid theoretical foundation, and then innovate and develop, and improve a patent’s value on the basis of previous patents.

Second, in-degree centrality is represented by the number of patent forward citations and reflects the extent to which a patent attracts subsequent innovators and competitors. This indicator not only reflects knowledge spillover but also the market value of companies. The results shows that in-degree centrality positively and significantly affects patent value, suggesting that when filing patents, a company should take into account how the patent’s unique technology could affect subsequent patents and attract more competitors. Such actions may increase the company’s higher reputation, improve its market value through competition in the sales market, and eventually increase patent value.

Moreover, other than redundancy in the network, effect size is the factor that reflects non-redundant information in the relationship between a patent and adjacent nodes. A smaller effect size of a patent indicates higher repeatability and closeness of the patent’s network. The results of this study indicate that effect size negatively and significantly affects patent value, suggesting that companies make their patents occupy a closed position of the network. This position can increase the opportunity to cooperate using similar techniques and can improve communication efficiency through homogeneous techniques to, as a result, create a closed network. Once the closed network is established, it will deepen R&D and the continuous improvements of existing techniques, lower the risk of technology development, and increase the value of a patent.

Companies can apply this model to evaluate the value of an opponent’s patents when selecting a target patent, and then obtain the right to use the patent through acquisition or authorization. Meanwhile, companies can also use social network analysis to build patent citation networks to detect technology development paths. Doing so will enable companies to identify the key patents in the development, and the positions in which the companies themselves and their opponents are located. Companies can then analyze and compare the layout strategies of themselves and their opponents to formulate appropriate competition and cooperation strategies.

Finally, this model provides companies with a set of early warning mechanisms for patent litigation. Using this model, companies can make judgments about the probability that their patents will undergo litigation. Because a patent citation network dynamically develops as time passes, companies are advised to dynamically update the patent database using this model. Doing this will enable companies to monitor changes in litigation probability in real time, identify in advance whether a patent may be involved in litigation, and prepare for patent litigation to reduce the significant cost and uncertainty related to future litigation.

## Supporting information

S1 Data The raw data of this paper. (XLSX)

S1 File Interview Guide. (DOCX)
