# Supplementary material for: Network characteristics and patent value—Evidence from the Light-Emitting Diode industry
Source: PLoS One. 2017 Aug 17;12(8):e0181988. doi: 10.1371/journal.pone.0181988 (PMC5560636; doi:10.1371/journal.pone.0181988)
Supplement: S1 File — (DOCX) [file pone.0181988.s002.docx]

**Supporting information 1: Interview guide**

To whom it may concern,

Greeting! We are patent value research group of Wuhan university, the purpose of this interview is to collect your opinions about light emitting diode (LED), thanks for your cooperation.

**Question：**

Q1: What keywords do you think are adequate to be searched in epitaxial growth of LED technology field?

Q2: What keywords do you think are adequate to be searched in chip making of LED technology field?

Q3: What keywords do you think are adequate to be searched in LED chip packaging of LED technology field?

Q4: Do you have any suggestion for this research interview?
